# The ABC recommendations for validation of supervised machine learning results in biomedical sciences

**DOI:** 10.3389/fdata.2022.979465

**Published:** 2022-09-27

**Authors:** Davide Chicco, Giuseppe Jurman

**Affiliations:** ^1^Institute of Health Policy Management and Evaluation, University of Toronto, Toronto, ON, Canada; ^2^Data Science for Health Unit, Fondazione Bruno Kessler, Trento, Italy

**Keywords:** supervised machine learning, computational validation, recommendations, data mining, best practices in machine learning

## 1. Introduction

Supervised machine learning has become pervasive in the biomedical sciences nowadays (Larrañaga et al., 2006; Tarca et al., 2007), and its validation has obtained a key role in all these scientific fields. We therefore read with great interest the article by Walsh et al. (2021), which reported a list of DOME recommendations to properly validate results achieved with supervised machine learning, according to the authors. In the past, several studies already listed common best practices and recommendations for the proper usage of machine learning (Bhaskar et al., 2006; Domingos, 2012; Chicco, 2017; Cearns et al., 2019; Stevens et al., 2020; Artrith et al., 2021; Cabitza and Campagner, 2021; Larson et al., 2021; Whalen et al., 2021; Lee et al., 2022) and computational statistics (Benjamin et al., 2018; Makin and de Xivry, 2019), but the comment by Walsh et al. (2021) has the merit to highlight the importance of computational validation, which is a key step perhaps even more important than the machine learning algorithm design itself.

Although interesting and complete, that article describes numerous of steps and aspects in a way that we find complicated, especially for beginners. We believe that the 21 questions of the Box 1 of the DOME article (Walsh et al., 2021) can be adequate for a data mining expert, but they might scare and discourage an inexperienced practitioner. For example, the recommendations about the *meta-predictions* and about the hyper-parameters' optimization might not be understandable by a machine learning beginner or by a wet lab biologist. And it should not be a problem: a robust machine learning analysis can be performed, in fact, without using meta-predictions or hyper-parameters, too. A beginner, in front of so many guidelines of that article, some of which being so complex, might even decide to abandon the computational intelligence analysis, to avoid making any mistake in their scientific project. Moreover, the DOME (Walsh et al., 2021) authors present the 21 questions of the article Box 1 with the same level of importance. In contrast, we think that three key aspects to keep in mind for computational validation are pivotal and can be sufficient, if verified correctly. So we believe that a practitioner would better focus all their attention and energy on accurately respecting these three recommendations.

**Figure 1:**
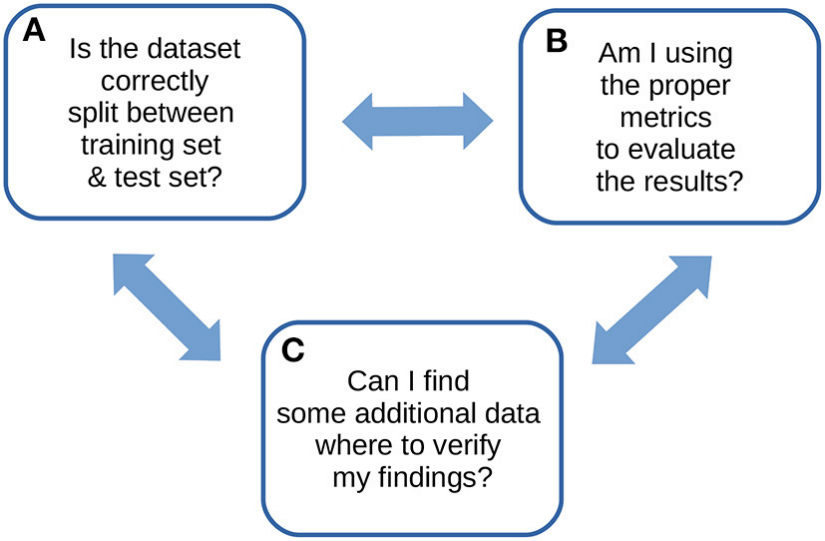
ABC recommendations checklist. An overview of our ABC recommendations, to keep in mind for any machine learning study.

We therefore wrote this note to propose our own recommendations for the computational validation of supervised machine learning results in the biomedical sciences: just three, explained easily and clearly, that alone can pave the way for a successful machine learning validation phase. We designed these simple quick tips from our experience gained on tens of biomedical projects involving machine learning phases. We call these recommendations ABC to highlight their essential role in any computational validation (Figure 1).

## 2. The ABC recommendations

### (A) Always divide the dataset carefully into separate training set and test set

This rule must become your obsession: verify and double-check that no data element is shared by both the training set and the test set. They must be completely independent.

You then can do anything you want on the training set, including the hyper-parameter optimization, but make sure you do not touch the test set. Leave the test set alone until your supervised machine learning model training has finished (and its hyper-parameters are optimized, if any). If you have enough data, consider also allocating a subset of it (such as 10% of data elements, randomly selected) as a holdout set (Skocik et al., 2016), to use as an alternative test set to confirm your findings and to avoid over-validation (Wainberg et al., 2016).

This important separation will allow you to avoid *data snooping* (White, 2000; Smith, 2021), that is a common mistake in multiple studies involving computational intelligence (Jensen, 2000; Sewell, 2021). Data snooping, also known as *data dredging* and called “the dark side of data mining” (Jensen, 2000), happens in fact when some data elements of the training set are present in the test set, too, and therefore over-optimistically improve the results obtained by the trained machine learning model on the test set. Sometimes, this problem can happen even when different data elements of the same patients (for example, radiography images in digital pathology) are shared between training set and test set, and is usually called *data leakage* (Bussola et al., 2021). This mistake is dangerous for every machine learning study, because it can give the illusion of success to an unaware researcher. In this situation, you need to keep in mind the famous quote by Richard Feynman: “The first principle is that you must not fool yourself, and you are the easiest person to fool” (Chicco, 2017).

Data snooping does exactly that: it makes you fool yourself and makes you believe you obtained excellent results, while actually machine learning performance was flawed. Once you make sure the training set and the test set are independent from each other, you can use traditional cross-validation methods such as *k*-fold cross-validation, leave-one-out cross-validation, and nested cross-validation (Yadav and Shukla, 2016), or bootstrap validation (Efron, 1992; Efron and Tibshirani, 1994), to mitigate over-fitting (Dietterich, 1995; Chicco, 2017). Moreover, over-fitting can be tackled through calibration methods such as calibration curves (Austin et al., 2022) or calibration-in-the-large (Crowson et al., 2016), which can also help measuring the robustness of model performance.

Moreover, it is important to notice that sometimes splitting the dataset into two subsets (training set and test set) might not be enough (Picard and Berk, 1990). Even for shallow machine learning models, a correct splitting methodology should be enforced: for instance, see the Data Analysis Protocol strategy introduced by the MAQC/SEQC initiatives led by the US Food and Drug Administration (FDA) (MAQC Consortium, 2010; Zhang et al., 2015). And when there are hyper-parameters to optimize (Feurer and Hutter, 2019), such as the number of hidden layers and the number of hidden units in artificial neural networks, it is advisable to split the dataset into three subsets: training set, validation set, and test set (Chicco, 2017). In these cases, sometimes in scientific literature the names *validation set* and *test set* are used interchangeably; in this report, we call *validation set* the part of the dataset employed to evaluate the algorithm configuration with a particular hyper-parameter value, and we call *test set* the portion of the dataset to keep untouched and eventually use to verify the algorithm with the optimal hyper-parameters' configuration.

### (B) Broadly use multiple rates to evaluate your results

Evaluate your results with various rates, and definitely include the Matthew's correlation coefficient (MCC) (Matthews, 1975) for binary classifications (Chicco and Jurman, 2020; Chicco et al., 2021a) and the coefficient of determination (*R*^2^) (Wright, 1921) for regression analyses (Chicco et al., 2021b). Moreover, make sure you include at least accuracy, *F*_1_ score, sensitivity, specificity, precision, negative predictive value, Cohen's Kappa, and the area under the curve (AUC) of the receiving operating characteristic curve (ROC) and of the prediction-recall curve (PR) for binary classifications. For regression analyses, make sure you incorporate at least mean absolute error (MAE), mean absolute percentage error (MAPE), mean square error (MSE), root mean square error (RMSE), and symmetric mean absolute percentage error (SMAPE), in addition to the already-mentioned *R*^2^. We recap our suggestions in Table 1.

**Table 1:** Recap of the suggested metrics for evaluating results of binary classifications and regression analyses.

**Analysis type**	**Always include**	**We suggest to include**
		TPR, TNR, PPV, NPV, accuracy,
Binary classification	MCC	F_1_ score, Cohen's Kappa,
		ROC AUC, and PR AUC
Regression analysis	R^2^	SMAPE, MAPE, MAE, MSE, and RMSE

The formulas of the binary classification rates can be found in Chicco and Jurman (2020) and Chicco et al. (2021a,c) and the formulas of the regression analysis rates can be found in Chicco et al. (2021b).

It is necessary to include all these scores because each of them provides a singular, useful piece of information about your supervised machine learning results. The more statistics you include, the more chances you have to spot any possible flaw in your predictions. All these rates work like dashboard indicator lamps in a car: if something somewhere in your machine (learning) did not work out the way it was supposed to, a lamp (rate) will inform you about it.

The Matthew's correlation coefficient, in particular, has a fundamental role in binary classification evaluation: it has a high score only if the classifier correctly predicted most of the positive elements and of the negative elements, and only if the classifier made mostly correct positive predictions and mostly correct negative predictions (Chicco and Jurman, 2020, 2022; Chicco et al., 2021; Chicco et al., 2021a). That means, a high MCC corresponds to a high score for all the four basic rates of a 2 × 2 confusion matrix: sensitivity, specificity, precision, and negative predictive value (Chicco et al., 2021a). Because of its efficacy, the MCC has been employed as the standard metric in several scientific projects. For example, the USFDA agency used the MCC as the main evaluation rate in the MicroArray II/Sequencing Quality Control (MAQC/SEQC) projects (MAQC Consortium, 2010; SEQC/MAQC-III Consortium, 2014).

Regarding regression analysis assessment, the coefficient of determination *R*-squared (*R*^2^) is the only rate that generates a high score only if the predictive algorithm was able to correctly predict most of the elements of each data class, considering their distribution (Chicco et al., 2021b). Additionally, *R*^2^ allows the comparison of models applied to datasets having different scales (Chicco et al., 2021b). Because of its effectiveness, the coefficient of determination has been employed as the standard evaluation metric for several international scientific projects, such as the Overhead Geopose DrivenData Challenge (DrivenData.org, 2022) and the Breast Cancer Prognosis DREAM Education Challenge (Bionetworks, 2021).

### (C) Confirm your findings with external data, if possible

If you can, use data coming from a different data source and made of a different data type from the main dataset to verify your discoveries. Obtaining the same results you achieved on the main original dataset on an external dataset coming from another scientific research centre would be a strong confirmation of your scientific findings. Moreover, if this external data were in a data type different from the original data, it would even increase the level of independence between the two datasets, and even more strongly confirm your scientific outcomes.

In a bioinformatics study, for example, Kustra and Zagdanski (2008) employed a data fusion approach to cluster microarray gene expression data and associate the derived clusters to Gene Ontology annotations (Gene Ontology Consortium, 2019). For validating their results, instead of using a different microarray dataset, the authors decided to take advantage of an external database made of a different data type: a protein–protein database called General Repository for Interaction Data Sets (GRID) (Breitkreutz et al., 2003). This way, the authors were able to find in external data a strong confirmation of the results they obtained on the original data, and therefore were able to claim their study outcomes as robust and reliable in their manuscript's conclusions.

Moving from bioinformatics to health informatics, a call for external data validation has recently been raised in machine learning and computational statistics applied to heart failure prediction as well (Shin et al., 2021).

That being said, we are aware that obtaining compatible additional data and integrating them might be difficult for some biomedical studies, but we still invite all the machine learning practitioners to make an attempt and to try to collect confirmatory data for their analyses anyway. In some cases, there are plenty of public datasets available for free use that can be downloaded and integrated easily.

Bioinformaticians working on gene expression analysis, for example, can take advantage of the thousands of different datasets available on the Gene Expression Omnibus (GEO) (Edgar et al., 2002). Tens of compatible datasets of a particular cancer type can be found by specifying the microarray platform, for example, through the recently released geoCancerPrognosticDatasetsRetriever (Alameer and Chicco, 2022) bioinformatics tool. Researchers can take advantage of these compatible datasets (for example, built on the GPL570 Affymetrix platform) to verify their findings, after applying some quality-control and preprocessing steps such as batch correction (Chen et al., 2011) and data normalization, if needed.

Moreover, public data repositories for biomedical domains, such as ophthalmology images (Khan et al., 2021), cancer images (Clark et al., 2013), or neuroblastoma electronic health records (Chicco et al., in press), can provide additional datasets that can be used as validation cohorts. Additional public datasets can be found on the University of California Irvine Machine Learning Repository (University of California Irvine, 1987), on the DREAM Challenges platform (Kueffner et al., 2019; Sage Bionetworks, 2022), or on Kaggle (Kaggle, 2022), for example.

When using external data, an aspect to keep in mind is checking and correcting issues like dataset shift (Finlayson et al., 2021) and model underspecification (D'Amour et al., 2020), which might jeopardize the coherence of the learning pipeline when moving from training and testing and validation.

## 3. Discussion

Computational intelligence makes computers able to identify trends in data that otherwise would be difficult or impossible to notice by humans. With the spread of new technologies and electronic devices able to save and store large amounts of data, data mining has become a ubiquitous tool in numerous scientific studies, especially in biomedical informatics. In these studies, the validation of the results obtained through supervised machine learning has become a crucial phase, especially because of the high risk of achieving over-optimistic, inflated results, that can even lead to false discoveries (Ioannidis, 2005).

In the past, several studies proposed rules and guidelines to develop more effective and efficient predictive models in medical informatics and computational epidemiology (Steyerberg and Vergouwe, 2014, Riley et al., 2016, 2021; Bonnett et al., 2019; Wolff et al., 2019; Navarro et al., 2021; Van Calster et al., 2021). Most of them however, provided complicated lists of steps and tips which might be hard to follow by machine learning practitioners, especially by beginners.

In this context, the article of Walsh et al. (2021) plays its part by describing thoroughly several DOME recommendations and steps for validating supervised machine learning results, but in our opinion it suffers from excessive complexity and might be difficult to follow by beginners. In this note, we propose our own simple, easy, essential ABC tips to keep in mind when validating results obtained with data mining methods.

We believe our ABC recommendations can be an effective tool to follow for all the machine learning practitioners, both by beginners and experienced ones, and can pave the way to stronger, more robust, more reliable scientific results in all the biomedical sciences.

## Author contributions

DC conceived the study and wrote most of the article. GJ reviewed and contributed to the article.

## Conflict of interest

The authors declare that the research was conducted in the absence of any commercial or financial relationships that could be construed as a potential conflict of interest.

## Publisher's note

All claims expressed in this article are solely those of the authors and do not necessarily represent those of their affiliated organizations, or those of the publisher, the editors and the reviewers. Any product that may be evaluated in this article, or claim that may be made by its manufacturer, is not guaranteed or endorsed by the publisher.
